# **The Healing Effect of Amniotic Membrane in Burn Patients**

**Published:** 2016-01

**Authors:** Mahdi Eskandarlou, Mina Azimi, Soghra Rabiee, Mohammad Ali Seif Rabiee

**Affiliations:** 1 Department of General Surgery, Hamadan University of Medical Sciences, Besat Hospital, Hamadan, Iran;; 2 Department of Obstetrics and Genecology, Hamadan University of Medical Sciences, Fatemieh Hospital, Hamadan, Iran;; 3 Department of Social Medicine, School of Medicine, Hamadan University of Medical Sciences, Hamadan, Iran

**Keywords:** Amniotic membrane, Burn, Healing

## Abstract

**BACKGROUND:**

Different methods for dressing of donor site of skin graft in burn patients have similarly pain, limitation of mobility of donor site and local complications such as infection and scar. Amniotic membrane has used for improvement of healing in some wounds. Accordingly in this study amnion was used as biologic dressing for donor site of skin graft to evaluate it’s efficacy in improvement of pain, move score and the risk of local infection.

**METHODS:**

Study was done as clinical trial over 32 admitted patients in burn department of Beasat hospital. Amnion was prepared in elective caesarean section after rule out any placental site for risk of torch and viral infection. Skin graft was taken from two sites in every patient. One site dressed with amnion and another with routine dressing. Then two sites were compared about severity of pain, move score, infection and time of dressing sloughing.

**RESULTS:**

Fourteen patients were women and 18 men. Mean score of pain and movement up to fourth and fifth post operative day respectively was less than control site. No difference is seen about infection and dressing slough in two sites.

**CONCLUSION:**

It seems use of amnion for dressing of donor site probably cause rapid epithelialisation and wound healing and can improve pain and move score in early post operative days. Accordingly it is expected to need less analgesia and low rate of immobilization and following complications and earlier discharge of patients.

## INTRODUCTION

Burn is still a devastating emergency with many physical and psychological disabilities^1^ and mortality and morbidity.^2^ Bacteria are important causes of nosocomial infection leading to septicemia and death in burn patients denoting to its public health importance.^3^ For survivors, scarring is still a problem posing psychological effects to the burn patient.^4^ Resistance to common therapies for several bacteria was noted in burn injuries complicating the situation more,^5^ even there have been efforts for treatment of burn wounds with medications having less adverse effects, such as medicinal herbals.^6^^-^^10^

Annually many individuals in low and middle income countries suffer from burn injuries.^11^ In the classic manner, the treatment of burn is via daily washing of the wound, removal of the dead tissue and antibiotic dressing till the formation of granulation tissue and later grafting.^12^ Different methods have been used for the care and dressing of the donor site due to the facilitation in the improvement and reduction of wound symptoms and therefore it implies the further possibility of donor site from the same locale.^6^^-^^10^^,^^13^^,^^14^ Among these the use of dry gas, gas dripping with an antibiotic, two or multilayer dressing, the vaseline gauze which is presently utilized in the scald department and amniotic membrane as biological dressing for treatment or care of some wounds can be mentioned.^15^^-^^18^ But yet, no any study described it’s using and efficacy for donor site of skin grafting. Therefore the present study was carried out with an aim to reveal the efficiency of the biological dressing with amniotic membrane for the reduction of pain in the donor site, improvement of the body movement in the donor site limb and evaluation of duration of dressing sloughing from the wound and risk of infection. 

## MATERIALS AND METHODS

This study was a clinical trial performed on 32 patients. These patients were hospitalized in the burn ward of the Besat Hospital due to burns and among the patients, the individuals aged over 18 years were enrolled. Each patient was considered as an intervention group and also as a control group. The one side of an organ was considered as an intervention area and the other side as a control region. 

The amniotic membrane in a fresh form was provided from the Department of Obstetrics and Gynaecology of Fatimane Hospital in an elective caesarean section. Infections to TORCH, hepatitis B, hepatitis C, AIDS, and syphilis before childbirth was not included. For hepatitis B and C, AIDS and syphilis infections, serological tests of HIVAb, HBS Ag, HCVAb and VDRL were carried out. 

After the cesarean section and under sterile conditions, the amniotic membrane was separated and placed in the normal saline solution and was immediately transferred to the burn department of the Apostolate Hospital in less than 6 hours from its isolation to be used for the study. The donor from the two limbs and or two harmonious body sections was selected using an electric dermatome with the thickness of 0.014 inch from each side. The dressing of the donor site in one side was done with vaseline gauze and was bandaged. 

For the dressing of the donor site on the opposite, the amniotic membrane was placed on the donor site and then the vaseline gauze on the membrane and then the dry gas was introduced and bandaged. The limbs that were subjected to study (i.e. the amniotic membrane placed on the donor site) was coded A, and the limb which was regarded as the control site (i.e. regular dressing or non-biological) was coded B. 

After the 24 hours of surgery, the bandage and the dry gases of both sites (case study and control) were removed till the level of vaseline gauze and the two sites were exposed to open air (hospitalized room) and later, the pain score, easiness in the move of organs, the spontaneous separation time of the last layer of dressing on the donor site and the condition of sepsis according to the protocol was provided. 

The pain score and easiness in the movement of the limbs that considered as the donor sites were determined by patient with the 1-5 and 1-10 score respectively in a daily examination from the patients and recorded in a questionnaire. Score 1 represented the lowest pain level and organ movement and 5 showed the highest organ movement level and 10 demonstrated the highest pain level and the validity of the infections in the two sites of case and control based on the daily clinical examination in the case of clinical symptoms based on the probability of sepsis (pain, discharge and inflammation, etc.), the cultivation was procured and in the case of bacterial growth over 100,000 colonies, it was considered positive. 

The patients who were discharged early post operatively, they were followed in the Sheikholraeis clinic and the required information about pain, organ movement, separation of dressings and sepsis were recorded in a questionnaire. The collected information from patients was eventually entered in the SPSS software (version 16, Chicago, IL, USA) and was analyzed using Mann Whitney test. In the context of investigating the presence of pain difference in various days in the donor sites, t-test was used till the fifth day and Mann Whitney test from the sixth day for the comparison of pain and easiness of the limbs movement scores.

## RESULTS

Thirty two patients with range of 18-88 years were enrolled while 18 patients (56%) were male. The pain status in the intervention and control areas were shown in Tables 1 and 2. The average pain score in the intervention areas was less than the control areas. From the 5^th^ to 14^th^ day, there was no significant difference in the pain score between two sides, although in all these cases, the pain score in the intervention area was less than the control area. The status of movement range in the intervention and control areas were demonstrated in Tables 3 and 4. 

**Table 1:** Comparing the average pain score in the two case area of intervention (dressing with amniotic membrane) and control (general dressing) in the varied research days

**Day**	**Average of intervention area**	**Number**	**Average of control area**	**Number **	**P value**
1	5.21	32	7.2	32	0.0001
2	4.15	32	5.9	32	0.0001
3	3.56	32	5.12	32	0.001
4	3.06	32	4.15	32	0.016
5	2.68	29	3.51	29	0.057

**Table 2:** Comparing the average pain ranks in the two case area of intervention (dressing with amniotic membrane) and control (general dressing) in the varied research days

**Day**	**Average of intervention area**	**Number**	**Average of control area**	**Number **	**P value **
6	19.69	21	23.31	21	0.32
7	16.17	18	20.83	18	0.17
8	9.55	10	11.45	10	0.44
9	6.25	6	6.75	6	0.79
10	5.3	5	5.7	5	0.81
11	3.5	3	3.5	3	1.00
12	1.75	2	3.25	2	PV0.22
13	1.75	2	3.25	2	PV0.22
14	1.5	1	1.5	1	1.00

**Table 3:** Comparing the average move score in the two case organs of intervention (dressing with amniotic membrane) and control (general dressing) in varied research days

**Day**	**Average of intervention area**	**Number **	**Average of control area**	**Number **	**P value**
1	3.06	32	2.03	32	0.0001
2	3.28	32	2.53	32	0.007
3	3.59	32	2.78	32	0.002
4	3.81	32	3.2	32	0.027
5	2.68	29	3.24	29	0.012

**Table 4:** Comparing the average move score in the two case organs of intervention (dressing with amniotic membrane) and control (general dressing in varied research days).

**Day **	**Average of intervention area**	**Number **	**Average of control area**	**Number **	**P value**
6	24.12	21	18.88	21	0.135
7	19.44	18	17.56	18	0.135
8	11	10	10	10	0.553
9	6.42	6	6.58	6	0.656
10	5.5	5	5.5	5	1.000
11	3.33	3	3.67	3	0.796
12	2.5	2	2.5	2	1.000
13	2.5	2	2.5	2	1.000
14	1.5	1	1.5	1	1.000

The movement condition in the intervention limbs till the fifth day of hospitalization in comparison to the control limb exhibited a significant difference (*p*=0.01) showing that the intervention cases which were dressed by the amniotic membrane had a better movement status in comparison to the routine dressing. From the 6^th^ day to the 14^th^ day, there was no significant difference between the movement of limbs in both sides, although in most of the cases, the intervention patients had a better movement status in relation to the control organ. 

The dressing separation time in the intervention cases from the days 3 to 18 after the surgery was about 8.15±3.4 days and in the control cases, the dressing separation in the days 5 to 21 was about 9.2±3.7 days while the difference of the dressing separation in intervention and control organs was not significant (*p*=0.29). Regarding the infection of intervention and control group, it was clear that two infection cases had appeared on the site dressed with amniotic membrane where in the general dressing site (control area), no infection was observed. The difference for infection in the dressings of intervention and control organs was not significant (*p*=0.49).

## DISCUSSION

After skin grafts, the donor site was painful and caused immobility of patients and also prone them to infection, hypertrophic scar formation and changes in color^19^ that can increase hospitalization period or even can be the indication for later cosmetic surgery. Immobility can cause deep vein thrombosis (DVT), and respiratory, gastrointestinal, endocrine and electrolyte disorders.^20^ Based on these complications after surgery especially skin transplantation, it is of great importance to prevent and reduce the complications few days after surgery. The coverage of donor site requires materials that preserve the epidermal function and integrate itself into the process of healing.^16^

Biological dressing was introduced as a gold standard for temporary covering of wounds.^21^ All biologic dressings are susceptible to early reaction and the only exception is the amniotic membrane.^21^ The use of amniotic membrane as biological dressing in the treatment of extensive burn wounds has been described in order to early recovery of patient, improvement of wound healing and its quality.^15^ Studies demonstrated that use of amnion in burn wounds can lead to reduction of pain intensity and prevent water and electrolyte disturbances and also can help for early preparation of the wound bed for grafting.^20^^,^^21^

The preparation and application of amniotic membrane according to our proposed method performed with simplicity and efficiency, lead to a significant difference for 3 variables between donor site with routine dressing and dressing with amniotic membrane. Biologic dressing lead to significant reduction of pain score at the site of skin graft in the first few days after surgery. In fact with covering of donor site with amnion, we introduced an occlusive dressing. Growth factors extravasated and accumulated in the interface of biologic dressing and abraded epidermis. This moisture and suitable interface could hasten the wound healing process and epithelialization.^16^

Amnion prevents the wound from irritation, evaporation, dryness and also nerve ending stimuli on the wound surface. These characters could not be seen in routine dressings. In the first 24-48 hours after any skin abrasion, skin harvesting or even primary repair in surgical wounds, basal epithelial cells can cause a water tight closure in wound layer together with migration and mitosis processes.^4^

This layer does not have enough strength against external mechanical stresses and is fragile. With disruption of this fine neo-epithelium, underlying nerve endings are exposed and suffered with contact to non-biological dressings. But after fifth day of graft taking, epithelialization reaches to optimal thickness and resistance in order to preserve its integrity against external stress. Therefore, pain score of both studied and control site did not show any difference after this time. Less pain at the first few days of skin harvesting is important. Patients could be mobilized soon and the need for analgesia during hospitalization is decreased and subsequent complications in respiratory and coagulation system are prevented.^16^

Early and feasible mobility of patients with application of amnion over the donor site seems to be a secondary effect of its pain subsiding. Although in this study, there was not statistical difference about the time of dressing separation for two types of dressing, but clinically the mean time of dressing sloughing in studied limb was 8.5 and for routine dressing was 9.2 days. This clinical difference is due to improvement of epithelialisation with amnion dressing while amniotic membrane has low antigenicity.^16^

Application of amnion in the first few days adheres tightly to wound surface and has positive modulation for wound healing process either in quality or in rapidity. Accordingly in this condition, possible morbidities of wound healing such as color change or hypertrophic scar would formation can be decreased. Dermal substitutes as biologic dressings can be used for wound care in plastic surgeries for this purpose but may not be cost-benefit.^16^ In this study, we did not have significant wound infection following to dressing by amnion. Amniotic membrane can prevent bacterial invasion to the donor site of wound by use of amniotic membrane. Similar conclusions could be seen in other studies.^17^^,^^22^

Reduction of pain severity and improvement of mobility with application of amniotic membrane over the donor site can cause earlier re-harvesting of graft from previous donor site specially in extensive burns and also decrease the duration of hospitalization, need for analgesia and complications of immobilization. Dressing of donor site of skin graft with amniotic membrane can reduce pain in the early post-operative days and facilitate the patient mobility and early sloughing of dressing from the wound bed. 

Due to ease of preparation and use of amniotic membrane as well as absence of adverse effects of its application, it is recommended to use amniotic membrane in the site of skin graft in order to be harvested in burn patients specially with high percentage of burn lesions for pain reduction and improvement of patient mobility and also acceleration of recovery of donor site for graft re-harvesting without any fear of infection. 
